# Treating depression at home with transcranial direct current stimulation: a feasibility study

**DOI:** 10.3389/fpsyt.2024.1335243

**Published:** 2024-03-04

**Authors:** Katharina Dragon, Mohamed A. Abdelnaim, Franziska C. Weber, Markus Heuschert, Leon Englert, Berthold Langguth, Tobias Hebel, Martin Schecklmann

**Affiliations:** ^1^ Department of Psychiatry and Psychotherapy, University of Regensburg, Regensburg, Germany; ^2^ University Medical Center, University of Regensburg, Regensburg, Germany

**Keywords:** non-invasive, transcranial direct current stimulation, tDCS, home treatment, feasibility

## Abstract

**Introduction:**

Treating major depressive disorder (MDD) with transcranial direct current stimulation (tDCS) devices at home has various logistic advantages compared to tDCS treatment in the clinic. However, preliminary (controlled) studies showed side effects such as skin lesions and difficulties in the implementation of home-based tDCS. Thus, more data are needed regarding the feasibility and possible disadvantages of home-based tDCS.

**Methods:**

Ten outpatients (23–69 years) with an acute depressive episode were included for this one-arm feasibility study testing home-based tDCS. All patients self-administered prefrontal tDCS (2 mA, 20 min, anodal left, cathodal right) at home on 30 consecutive working days supported by video consultations. Correct implementation of the home-based treatment was analyzed with tDCS recordings. Feasibility was examined by treatment compliance. For additional analyses of effectiveness, three depression scores were used: Hamilton depression rating scale (HDRS-21), Major Depression Inventory (MDI), and the subscale depression of the Depression-Anxiety-Stress Scale (DASS). Furthermore, usability was measured with the user experience questionnaire (UEQ). Tolerability was analyzed by the number of reported adverse events (AEs).

**Results:**

Eight patients did not stick to the protocol. AEs were minimal. Four patients responded to the home treatment according to the MDI. Usability was judged positive by the patients.

**Conclusions:**

Regular video consultations or other safety concepts are recommended regardless of the number of video sessions actually conducted. Home-based tDCS seems to be safe and handy in our feasibility study, warranting further investigation.

## Introduction

Transcranial direct current stimulation (tDCS) is a non-invasive brain stimulation (NIBS) technique that induces a weak constant direct current (1–2 mA) via electrodes that are placed on the scalp. Thus, cortical excitability can be modulated by changing the resting membrane potential (1). Treatment over several weeks has the potential to alter pathological cortical plasticity in various psychiatric diseases (2). Conventionally, a tDCS device has an anodal electrode, which increases the excitability of the underlying cortex, and a cathodal electrode, which decreases the excitability of the underlying cortex (1). For treating major depressive disorder (MDD), the anode is placed over the left dorsolateral prefrontal cortex (DLPFC) and the cathode is placed over the right DLPFC (3). The rationale for investigating tDCS as a treatment for depression is based on considerations of hypometabolism of the left DLPFC and right prefrontal hypermetabolism as well as dysfunction of brain plasticity, characterized by an alteration of long-term potentiation for depression (4). Thus, by simultaneously increasing the neuronal activity on the left and decreasing the activity on the right side of the DLPFC, antidepressant effects can be achieved (5). A meta-analysis by Razza et al. (6) has already shown that the effects of active tDCS are superior to sham conditions, but with rather small to medium effect sizes. Furthermore, Brunoni et al. (7) have shown that therapeutic effects of tDCS may be mediated by pharmacological modulation of neurons associated with depression in deep brain structures, although they are not directly affected by superficial current flow generated by tDCS stimulation. Nevertheless, tDCS is a promising therapy option for more than one-third of patients who do not achieve remission after multiple treatment trials (7, 8).

To date, tDCS treatment is typically applied at a medical facility by trained medical staff (3). However, daily preparation and the application of the tDCS stimulation itself (20–30 min) take time and staff capacity (9). Daily arrivals at the clinic require additional resources and limit its applicability for patients living far away from a treatment center.

Home-based tDCS treatment has been proposed and investigated for several years (3) as tDCS devices are small, portable, and relatively low-cost and have a favorable side effect profile. Specific devices for home treatment were developed that can be programmed in the clinic beforehand so that patients can use them at home just by activating the stimulation device (9). Although antidepressant treatment at home is possible with a portable tDCS device, an implementation at home can have some disadvantages, like incorrect placement of the electrodes or the risk of overstimulation (10). In order to minimize such adverse events (AEs) and to ensure correct training and supervision of the patients, the first measurement in our study was carried out at the hospital. Additionally, all patients received a comprehensive introduction to the device. Another disadvantage that might come with home-based treatment is the lack of contact with researchers, which might positively impact depressive symptoms due to social interaction (11). In order to ensure contact nonetheless and to supervise regular implementation and the documentation of possible side effects (headaches, etc.) regular video calls with medical staff were implemented. Although patients using home-based tDCS no longer have any travel costs, the use of accessible home-based tDCS devices is still costly due to license fees for tele-therapy; room costs (heating, etc.), data protection processing programs, and staff workload are still comparable for treatment in a clinic (12).

Nevertheless, deploying tDCS treatment at home comes with many advantages, such as reaching more patients (13). Moreover, outpatients who suffer from a depressive episode with pronounced avolition are not required to travel to the clinic for daily treatment (3). Additionally, given the COVID-19 pandemic in which frequent personal contact was avoided anyway, depression treatment with NIBS could take place continuously (14, 15). Hence, the number of studies concerning tDCS home-based treatment for depression is increasing (13). According to the review by Kumpf et al. (9), to date, nine previous studies that primarily targeted home-based tDCS on depressive symptoms of 231 patients have shown a trend towards good antidepressant effectiveness, i.e., amelioration of symptoms in uncontrolled trials. According to Woodham et al. (16), in an open-label trial of 4 weeks, Alonzo et al. (3) found a response rate of 38% (*n* = 33) and Borrione et al. (17) found a response rate of 80% (*n* = 5) using a tDCS protocol combined with an app-based psychological intervention. Most of the few sham-controlled studies have not found a significant difference between active and placebo stimulation so far [Mota et al. (18), Lee et al. (19)]. One sham-controlled home-based tDCS trial by Oh et al. (20) has found a significant difference between active and sham tDCS, but 13/58 participants did not complete the study and all participants were additionally prescribed escitalopram 5–20 mg/day. Furthermore, Kumpf et al. (9) have shown that home-based tDCS trials for depression vary strongly in treatment parameters such as electrode positioning, current intensity, or number of sessions. Thus, more research regarding the implementation of home-based tDCS treatment for depression is needed (9).

Here, we conducted a one-arm feasibility study to determine the feasibility of video monitoring and related tDCS parameters of a 6-week home-based tDCS treatment for patients suffering from MDD. Additionally, we investigated clinical outcome measures. The time frame of 6 weeks was chosen because similar in-clinic protocols yield the best effects (10).

## Methods and materials

### Subjects and study design

The study protocol, patient information, and consent forms were approved by the local ethics committee of the University of Regensburg (20-2091-101). The trial was registered at the U.S. National Institutes of Health Database (www.clinicaltrials.gov) accessible with the identifier code NCT05123872. All patients gave written informed consent to the study. Recruitment took place via a pool of outpatients of the Bezirksklinikum Regensburg (Germany) and via outpatients of psychotherapists of Regensburg. Outpatients of both sexes were eligible for the study if they (1) were aged 18–70 years, (2) suffered from a depressive episode relating to unipolar or bipolar depression as identified by ICD-10 criteria (21) and/or (3) had a score of at least 21 points in the 21-item Hamilton depression rating scale (HDRS 21), (4) had stable psychotropic medication for at least 2 weeks, and (5) had internet connection at home and used the provided video -call set-p. Exclusion criteria were (1) contraindication for treatment with tDCS (e.g., electronic implants, cardiac pacemakers, or dermatological diseases), (2) neurological diseases (e.g., history of seizures), (3) simultaneous participation in a different study, and (4) pregnancy or lactation period.

Ten outpatients were recruited from fall 2020 to fall 2021. For a better overview, the original numbering was maintained (Pat 1–10). One male patient was treated erroneously with a current of only 1 mA (Pat 2, see below) and was therefore excluded from further analyses. Thus, we recruited one additional male patient in spring 2023 (Pat 11). Additionally, because of more than 50% missing data and delayed return of the tDCS device, data from one female patient (Pat 10, see below) had to be excluded from analyses.

**Figure 1:**
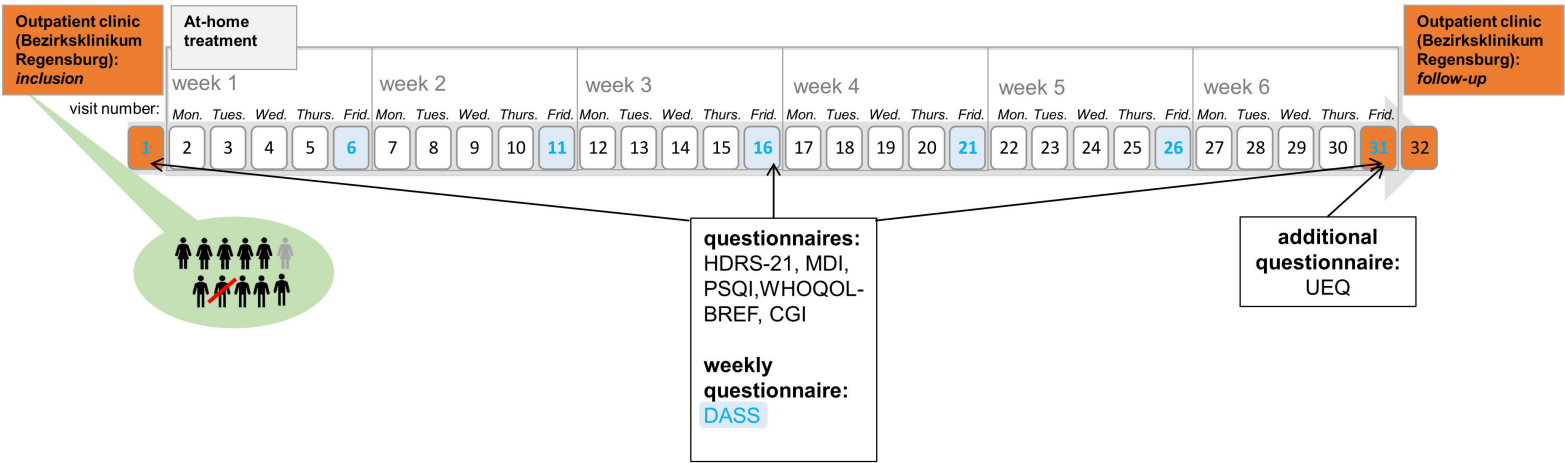
Course of the trial. This figure shows the course of the study. Inclusion, visit 31, and visit 32 (follow up measures) took place in the hospital (orange). Home-based treatment is depicted in gray. The Depression-Anxiety-Stress Scale was completed every Friday (blue).

At baseline, week 3, and week 6 (end of treatment), the severity of depressive symptoms was assessed with three different questionnaires as not to miss any possible effects on different clinical aspects. Observer-based ratings were assessed with the 21-item Hamilton Depression Rating Scale (HDRS-21; 22), which scores from 0 to 66. Self-reported symptoms were assessed with the Major Depression Inventory (MDI; 23), which scores from 0 to 50. Weekly surveys were covered with the Depression-Anxiety-Stress Scale (DASS; 24). Here, we focused on the changes in the depression subscale, which scores from 0 to 21. In all three questionnaires, higher scores indicate more depressive symptoms. At week 3, the HDRS-21 was assessed via video consultation (see below). For additional analyses, patients completed at these time points the Pittsburgh Sleep Quality Index (PSQI; 25) and an abbreviated version of the WHO quality-of-life scale (WHOQOL-BREF; 26), which is divided into four domains: physical health (domain 1), psychological health (domain 2), social relationships (domain 3), and environment (domain 4). In order to investigate the subjective impression of the users toward home treatment, patients completed at week 6 the user experience questionnaire (UEQ), which is based on the open-source evaluation method by Schrepp et al. (27). The questionnaire is divided into six scales, which measure the classical usability aspects as well as user experience aspects. The six scales include Attractiveness (Overall impression of the product), Perspicuity (Is it easy to get familiar with the product)?, Efficiency (Can users solve their tasks without unnecessary effort)?, Dependability (Does the user feel in control of the interaction)?, Stimulation (Is it exciting and motivating to use the product)?, and Novelty (Is the design of the product creative)? (https://www.ueq-online.org/; access: 2024-01-30). Higher UEQ scores correspond to better evaluation. Additionally, clinicians completed the seven-level scale Clinical Global Impression Scales (CGI-Severity and CGI-Improvement; 28) for quantifying and tracking the patient’s treatment response over the course of the trial (see **Figure 1**).

### tDCS: home treatment

Two hospital visits were mandatory for study participation: one pre-treatment and one post-treatment (**Figure 1**). The initial visit, conducted at the Bezirksklinikum Regensburg outpatient clinic, involved both the first tDCS treatment and comprehensive patient training for home sessions. A medical technical assistant meticulously instructed participants on electrode placement and treatment protocol, ensuring accurate and safe self-administration upon discharge. During the subsequent at-home phase, adherence and treatment safety were monitored via daily video consultations, facilitated by the CLICKDOC software (version 5.9.1, La-Well Systems GmbH), a clinically approved platform. These consultations verified proper electrode placement, confirmed treatment initiation, and monitored for any AEs that were noted on a treatment protocol. Only participants demonstrating consistent adherence and correct electrode positioning without any further instruction needed were permitted to undergo unsupervised treatment sessions as long as they did not report any side effect in the first five consecutive sessions. Treatment parameters remained consistent throughout the study: On 30 consecutive weekdays with video consultations once a day, each session delivered a 2-mA current for 20 min using a prefrontal montage. The CE-certified DC-Stimulator Mobile (Neuroconn, Ilmenau, Germany) was employed for all stimulations and could be activated by the study participants at any time.

**Figure 2:**
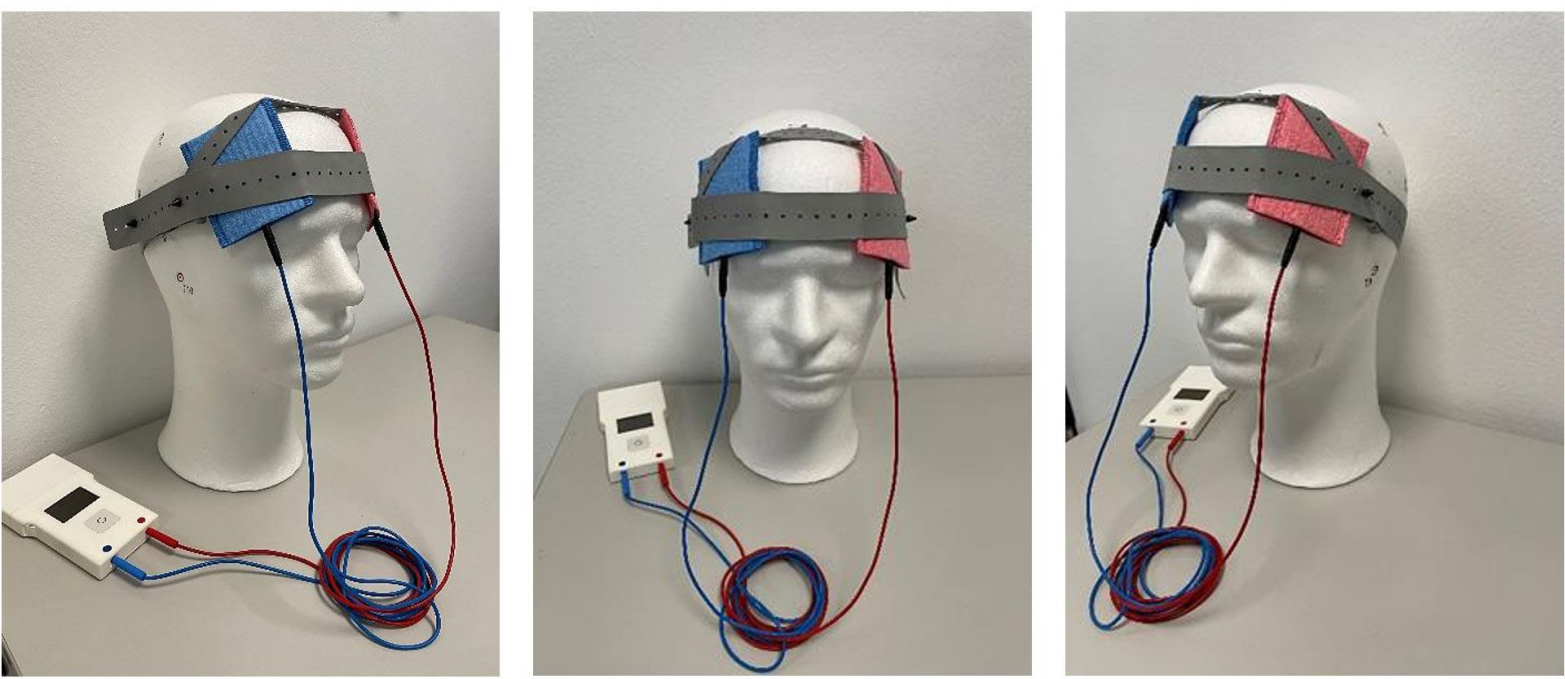
Prefrontal setup of the tDCS device for home treatment.

At the initial visit, participants received a personalized tDCS kit composed of the stimulation device, two 5×7 cm rubber electrodes, color-coded sponges (anode: red, cathode: blue), NaCl 0.9% solution for sponge soaking, and an instruction manual with detailed illustrations. This standardized protocol, coupled with daily monitoring and adherence checks, aimed to ensure the safety of home-based tDCS treatment for all participants (see **Figure 2**).

### Statistical analysis

We used descriptive statistics to summarize the clinical and demographic characteristics of the sample and the completion rates.

Since we focused on the tDCS treatment outside of a clinical setting, we analyzed the tDCS data (regularity of implementation without video consultation, number of video consultations, etc.) as primary outcome. For this purpose, the tDCS recordings (mean amperage, mean voltage, mean time of treatment, etc.) were extracted from the output neuroConn LogFiles and mean scores were calculated within Microsoft Excel. Subjective rating of the treatment usability (UEQ) was analyzed as primary outcome, on a descriptive level. Any AEs that occurred were coded if reported at any intensity or duration. AE occurrences were estimated by the number of participants reporting an AE in at least one of their tDCS sessions.

In accordance with our registry at clinicaltrials.gov (see above), all depression questionnaires (HDRS-21, MDI, and DASS) were also defined as primary outcome measures. The number of responders was defined by ≥50% reduction in the mean scores after the treatment duration of 6 weeks (efficiency) according to the HDRS-21. Collected follow-up data (after 18 weeks, visit 32) was not further analyzed due to >50% missing data. Thus, the planned secondary outcome measures (changes of the HDRS-21, MDI, CGI-I, PSQI, WHOQOL-BREF, and DASS between baseline and week 18) could not be calculated. Accordingly, outcome measures were calculated for a time frame of 6 weeks. Thus, repeated-measures analysis of variance (ANOVA) with time as within factor (three levels: baseline vs. week 3 vs. week 6) were used for the estimation of secondary treatment effects. For the weekly DASS data, another ANOVA for the subscale *depression* with time as within factor (seven levels: baseline vs. week 1 vs. week 2 vs. week 3 vs. week 4 vs. week 5 vs. week 6) was calculated, despite 44% missing data for this questionnaire. Subsequent paired samples *t*-tests were calculated for *post-hoc* analyses. Regarding the feasibility, both patient reports and log files of the used tDCS devices were analyzed. All 10 patients are listed corresponding to the time of the first treatment. All statistical analyses were conducted with SPSS version 28.0 (IBM SPSS, Chicago, IL).

Due to the use of three depression measurements, threshold level of significance was adjusted for multiple comparisons by Bonferroni’s correction (*p* = 0.017). Mean (M) and standard deviation (SD) are reported. The mean values of the PSQI and WHOQOL-BREF were conducted with Microsoft Excel sheets. The mean values of the UEQ were analyzed with Microsoft Excel (ueq-online.org), by Schrepp et al. (27).

## Results

### Demographics

All patients suffered from an acute depressive episode (ICD-10: F32.1, F33.1, and F33.2). Our sample consisted of one full-time employee, two half-time employees, three students, one early pensioner, one pensioner, and two unemployed patients. Two patients were single, and eight were in a relationship. Eight patients were high school graduates, one patient did not have an academic degree, and one educational information was missing.

Further demographic and clinical characteristics of the enrolled patients are provided in **Tables 1**, **2**.

**Table 1:** Clinical data at baseline of the present sample.

General variables	
Age: M (SD)	37.40 (14.14)
Age: range	23 - 69
Gender: m/f (*N*)	4/6 (10)
Questionnaire scores at baseline: M (SD)	
HDRS-21(0–65)	18.90 (4.04)
MDI (0–50)	32.50 (6.54)
DASS, depression subscale (0–12)	11.25 (4.92)
WHOQOL-BREF physical health subdomain (4–20)	11.77 (3.22)
WHOQOL-BREF psychological subdomain (4–20)	10.43 (1.56)
WHOQOL-BREF social subdomain (4–20)	12.67 (2.61)
WHOQOL-BREF environment subdomain (4–20)	14.60 (2.01)
PSQI total sum (0–21)	8.78 (5.49)
CGI (1–7)	4.44 (.53)

Questionnaire scores at baseline were calculated without patient 10. HDRS-21, Hamilton Depression Scale 21 items. MDI, Major Depression Inventory. WHOQOL-BREF, World Health Organization Quality of Life Questionnaire short version; higher scores indicate better quality of life. PSQI, Pittsburgh Sleep Quality Index. CGI, Clinical Global Impression; ordinal scale.

**Table 2:** Clinical data per patient.

Patient	Comorbid psychiatric diagnoses (ICD-10)	Comorbid diseases	Psychiatric medication (dosage)
**1**		Tinnitus	Escitalopram (10 mg), tebonin (120 mg), doxepin (20 mg)
**3**	Borderline personality disorder (F60.31), ADHD (F90.0), adjustment disorder (F43.2)		sertralin (100 mg), olanzapin (7.5 mg), sumatriptan (100 mg)
**4**			
**5**	ADHD (F90.0)		Atomoxetin (60 mg)
**6**			
**7**			Agomelatin (u.d.)
**8**			Trimipramin (25 mg)
**9**			Escitaloptam (5 mg)
**10**		Hypothyreosis	
**11**		arterial hypertension, arthosis	Venlaflaxin (75 mg), hydrochlorothiazide (20 mg), zanipress (20 mg)

ADHD, attention deficit/hyperactivity disorder. u.d.: unknown dosage. Patients were taking daily antidepressants. Doses were not changed throughout the study.

### Feasibility

All participants performed an average of 29.6 stimulation sessions over the course of 6 weeks (**Table 3**). Most of the patients conducted the treatments in the morning or at noon. Eight patients did not stick to the protocol, meaning that according to the tDCS log files, some patients conducted the treatment not only on working days but also, for example, even on holidays or on weekends. For example, patient 11 conducted the treatment every day. In four patients, the time of treatment was highly variable: patient 4 underwent treatment between 7:16 a.m. and 10:57 p.m. and patient 7 underwent treatment between 7:30 a.m. and 4:16 p.m. Patient 10 underwent treatment three times at night. Patient 11 underwent treatment at 7:00 a.m. during supervision and at 7:00 p.m. without supervision. Only two participants underwent the treatment regularly at the same time as instructed.

**Table 3:** Mean values for the tDCS data.

Patient	Amperage (mA)	Electrical voltage (V)	Average time of treatment	Days of treatment	Days without supervision
1	1.981	4.565	11:44 a.m.	28	0
3	1.982	5.308	12:43 p.m.	29	1
4	1.979	4.926	12:16 p.m.	28	10
5	1.993	4.657	7:58 a.m.	31	5
6	1.982	5.605	1:29 p.m.	28	3
7	1.985	5.321	11:16 a.m.	30	4
8	1.980	4.766	12:47 p.m.	34	3
9	1.979	6.277	9:06 a.m.	29	2
10	1.982	5.115	8:06 a.m.	23	9
11	1.982	6.416	3:32 p.m.	36	22

Days of treatment include first visit at hospital. Patients 10 and 11 ended two treatments a few seconds before the regular ending of the stimulation after 20 min. Patient 11 had to restart the treatment 11 times due to “cancellation by error” by the device itself.

### Usability and tolerability

**Figure 3:**
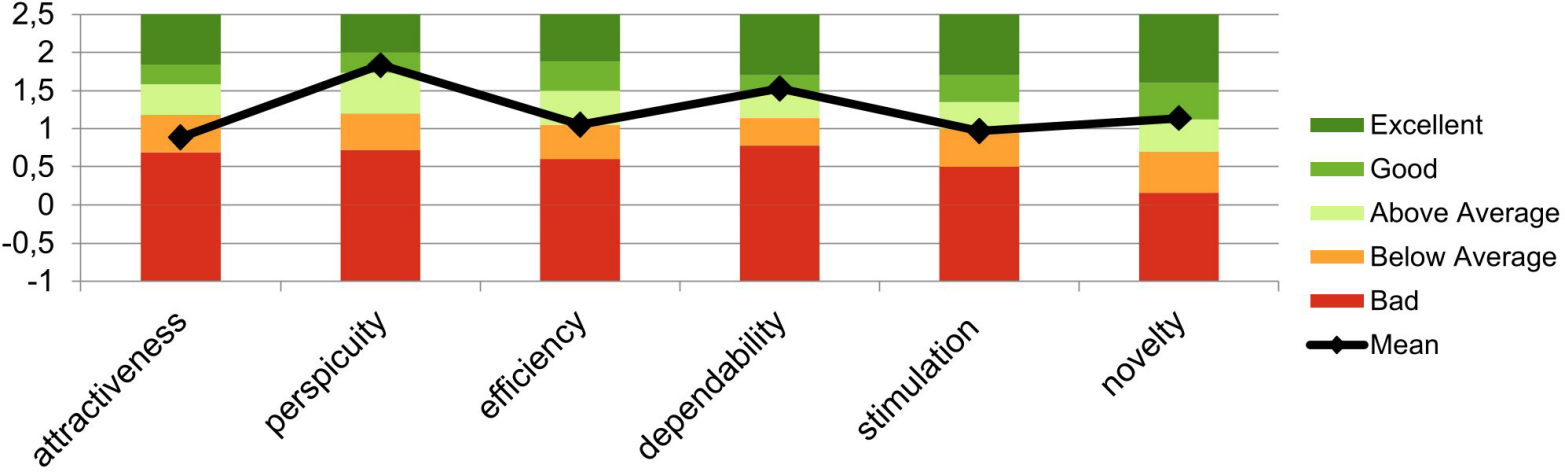
Mean evaluation of the user experience based on the evaluation method by Schrepp et al. (27). This graph shows the mean scores and SDs of the six factors of the user experience questionnaire (UEQ) across the sample.

Based on the evaluation method by Schrepp et al. (27), all patients evaluated the treatment as follows: the scales *Attractiveness* (M = 0.89, SD = 0.81) and *Stimulation* (M = 0.97, SD = 0.93) were rated “below average”. *Efficiency* was rated “above average” (M =1.06, SD = 0.79). The scales *Perspicuity* (M = 1.83, SD = 1.56), *Novelty* (M = 1.14, SD = 0.38), and *Dependability* (M = 1.53, SD = 0.85) were rated “good” (**Figure 3**).

No serious AEs occurred in any of the patients. Side effects were noted as free text by the medical staff on the treatment protocol: 2/10 patients indicated mild headaches after treatment during the first week. One patient felt tingling over the course of the entire treatment. Another patient felt tingling during the first week of treatment. One of ten patients indicated mild redness on the left side of his head after treatment 5 and 6. The number of side effects was not related to the number of sessions.

### Additional analyses: effectiveness

Each level of all within-subjects factors, regarding the HDRS-21 and MDI data, was approximately normally distributed, as assessed by the Shapiro–Wilk test, *p* > 0.05.

**Table 4** provides all results concerning depression measurements in the course of the trial. There was a statistically significant reduction (change in %) of the mean MDI scores after treatment compared to baseline ( $\frac{\left(\text{week }6-\text{Baseline}\right)}{\text{Baseline}}$ ). In contrast, no significant reduction of the mean HDRS-21 scores was found.

**Table 4:** Sum scores for all participants over the course of the trial for two of the depression measurements.

	BL	Week 3	Week 6	Change (%)	95% CI lower	95% CI upper	
HDRS-21							
Pat 1	14.00	15.00	21.00	+50.0			
Pat 3	27.00	15.00	9.00	−66.6			
Pat 4	18.00	14.00	12.00	−33.3			
Pat 5	17.00	6.00	8.00	−53.0			
Pat 6	19.00	16.00	18.00	−5.3			
Pat 7	16.00	16.00	22.00	+37.5			
Pat 8	22.00	12.00	11.00	−50.0			
Pat 9	22.00	20.00	5.00	−77.3			
Pat 11	14.00	-99	7.00	−50.0			
**Total (SD)**	19.38 (4.14)	14.25 (4.03)	13.25 (6.32)		13.76	17.68	*F *(2,14) = 3.13, partial η² = 0.309, *p* = 0.075
MDI							
Pat 1	20.00	19.00	16.00	−20.0%			
Pat 3	39.00	32.00	15.00	−61.5%			
Pat 4	32.00	34.00	25.00	−21.9%			
Pat 5	25.00	8.00	8.00	−68.0%			
Pat 6	41.00	37.00	36.00	−12.2%			
Pat 7	37.00	30.00	27.00	−27.0%			
Pat 8	37.00	26.00	20.00	−45.9%			
Pat 9	34.00	5.00	6.00	−82.4%			
Pat 11	31.00	25.00	7.00	−77.4%			
**Total (SD)**	32.89 (6.81)	24.00 (11.27)	17.78 (10.22)		18.53	31.24	*F *(2,16) = 14.28, partial η² = 0.641, *p*< 0.001

Responders are shown in orange. BL, Baseline. +: increase in the depression measurement (%). −99: missing data. SD are shown for the present sample.

Five participants responded to the treatment confirmed by the HDRS-21, corresponding to 55.5% of the sample. Four of these five participants additionally responded to the treatment confirmed by the MDI, which corresponds to 44.4% of the sample (see **Table 4**).

Concerning the MDI scores, *post-hoc* analyses revealed significant reductions after week 3 [*t*(8) = 2.86, *p* = 0.021] and week 6 [*t*(8) = 5.03, *p* = 0.001] compared to baseline.

**Figure 4:**
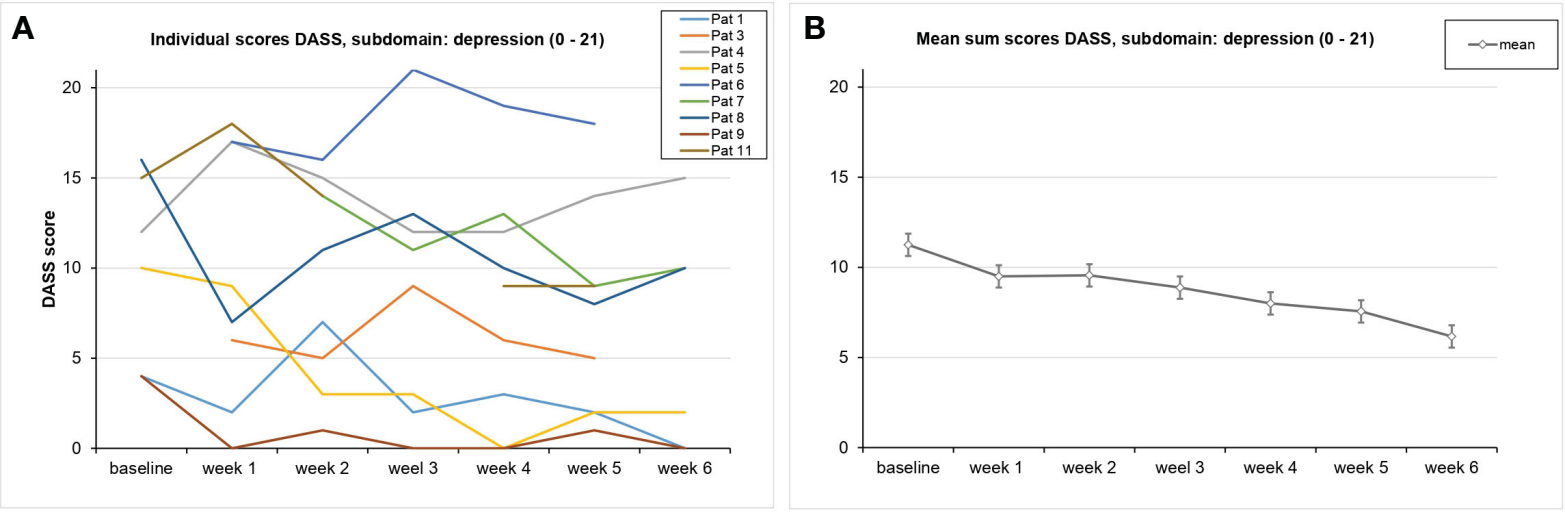
Course of the DASS values on average **(A)** and for each patient **(B)** This graph shows the DASS scores for the subscale *depression* over the course of the trial for each participant. SDs are not plotted for presentational purposes. Missing values are not replaced. Responders are shown in orange.

Because of high correlations among the seven measurements concerning the DASS data, the Greenhouse–Geisser correction was used: Over the course of the trial, no significant reduction in the depression subscale of the DASS was found [*F*(2.5,10.2) = 1.89, *p* = 0.197, partial η² = 0.321] (**Figure 4**). In analyses of the DASS data, there were four responders: Pat 5 (−80%), Pat 9 (−100%), Pat 8 (−80%), and Pat 1 (−100%).

**Figure 5:**
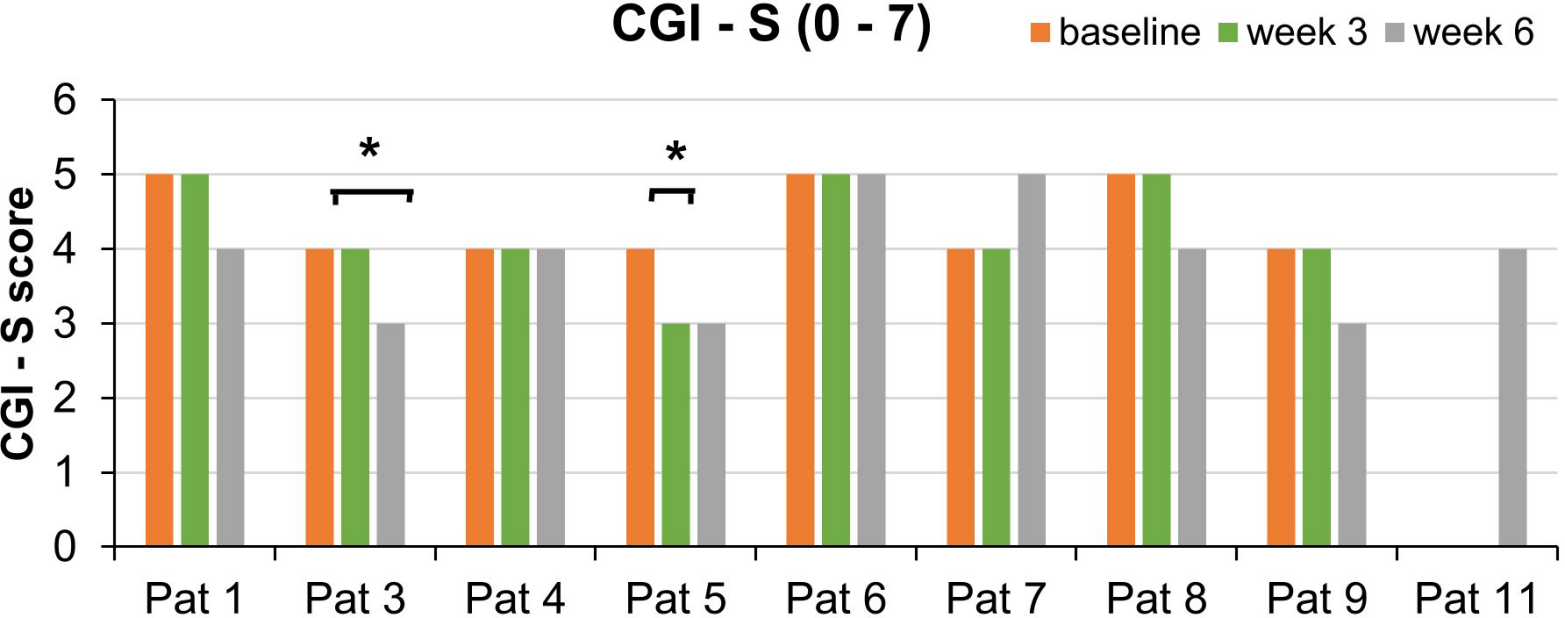
Course of the CGI-S scores for each patient split by week 3 and week 6. This bar graph shows the CGI-S scores over the course of the trial. *Significant improvement of the global impression as measured by the CGI-I (score: 2). SDs are not plotted for presentational purposes. Missing values are not replaced.

Regarding the CGI-I measurements, equivalent improvements were found for two of the same patients: The third patient’s illness was estimated as improved (score: 2) by the clinicians after week 6. Patient 5 was considered as improved already after week 3 (**Figure 5**).

**Figure 6:**
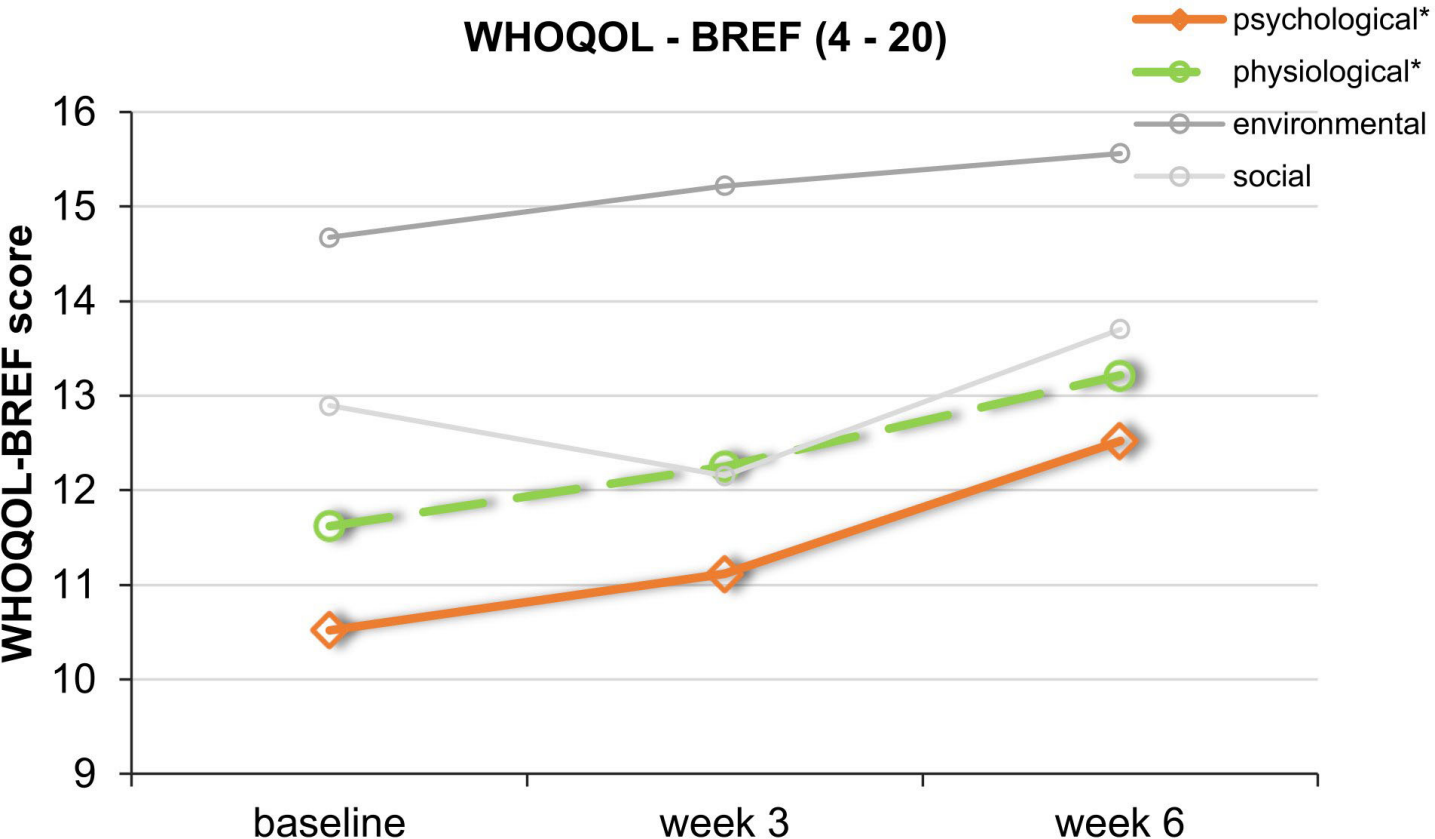
Course of the mean values of the 4 subdomains of the WHOQOL-BREF. This graph shows WHOQOL-BREF mean scores over the course of the trial. *Significant improvement for the physical (green) and psychological (orange) subdomains. SDs are not plotted for presentational purposes.

Repeated-measures ANOVAs for secondary outcome measures revealed no significant improvement of the patients sleep regarding the PSQI scores. However, there was a statistically significant improvement in the psychological subdomain of the WHOQOL-BREF over the course of the study [95% CI: 9.53 to 13.23; *F*(2,16) = 4.71, *p* = 0.025, partial η² = 0.371]. In the physical health subdomain, there was also a statistically significant increase in quality of life [95% CI: 9.35 to 15.37; *F*(2,16) = 3.76, *p* = 0.046, partial η² = 0.320]. Regarding the social and environment subdomain, there were no significant changes (**Figure 6**) (*p*s > 0.157).

## Discussion

The present one-arm feasibility study investigated in a small sample of patients different aspects regarding tDCS treatment of depression at home. The study revealed possible difficulties in carrying out a tDCS treatment outside of a clinical setting (e.g., tertiary care hospital), despite the support of regularly planned video consultations. Additionally, we provide further data regarding clinical outcome measurements for home-based tDCS treatment.

Although we had the impression with a few participants that after a certain number of monitored sessions they could perform the tDCS treatments on their own, we found from analyzing the tDCS data recordings that more than half of the study participants did not adhere to the pre-discussed treatment protocol (e.g., treatment on holidays or weekends). As an example, patient 10 forgot the treatment (and video consultations) three times and performed the stimulation at nighttime without supervision and with a short interval to next day’s treatment. Thus, if certain patients failed to attend the scheduled video call, then they performed the treatment without video supervision and presumably not entirely correctly. Further results showed that patients 3, 5, and 11, all treatment responders, restarted the treatment on their own if there was any problem with the tDCS device or if their recording was uncompleted. This highlights the need for daily video calls to check the correct implementation or that the device is programmed in a way that it can only be switched on at a certain time, because even though those 3 patients were able to restart the stimulation on their own and completed the treatment correctly, it highlights the risk of overstimulation for incautious patients. Previous studies have shown that the number and interval of sessions are critical concerning safety. With higher numbers of sessions and shorter intervals, the risk of side effects increases (29, 30). In our study, one patient had a comorbid borderline personality disorder that comes with a high risk of self-harming behavior (31). As already stated in a review by Kumpf et al. (9), regular supervision of home-based treatment and technical control of the device are important in order to minimize possible side effects and risks of deliberated self-harm. Our results show that a home-based tDCS device has to be remotely adjusted, because although the clinicians in this study saw no need for real-time video consultation, there were some subjects who did not adhere to the protocol. Future studies should consider a security system to permit daily use for 20 min with a minimal interval of 12 h between sessions, as, e.g., in Carvalho et al. (12). The use of pre-programmed home-based tDCS would allow patients to choose what time of day to receive the treatment, therefore accommodating patients’ schedules and minimizing possible side effects. Future studies with real-life video consultations should at least consider a fast-track contact line. This would ensure that patients could report any side effects or get help with technical problems. Another option would be to resort to daily written feedback to clinicians, which would allow them to decide whether to contact respective patients. Although we only found minimal deviations from the protocol, future studies should ensure that patients comply to agreed arrangements.

Furthermore, our results show that for patient 2, the device was incorrectly set, because he was stimulated with a very low mean amperage of 0.995 mA. This problem was only detected after study finalization. This highlights the need for corresponding training of the instructing staff. A recent investigation concerning another NIBS, home-based tES (transcranial electrical stimulation), showed that an educational program for remote training and supervision at home could facilitate further research (32).

Our study participants evaluated the treatment as not hard to learn (UEQ factor: Perspicuity). However, the overall impression of the product (UEQ factor: Attractiveness) and the excitement/motivation to use the product (UEQ factor: Stimulation) were both rated low (“below average”). Overall, home-based tDCS seems to be moderately user-friendly when using the Neuroconn home-based tDCS system the way this study did. According to previous studies, we registered no serious adverse effects and only few minor side effects (subjective sensations of tingling or headaches/pain during the first treatments and/or mild skin redness), confirming that tDCS is a tolerable treatment method (13)—even at home. This is in contrast to a recent published study by Kumpf et al. (9), where their home-based trial had to be prematurely terminated due to an accumulation of skin lesions. These findings highlight the need for careful and active side effect monitoring before and after stimulation, e.g., in the form of a safety questionnaire, as MDD patients may be impaired in their ability to proactively report side effects (9).

As this one-arm study did not include a control condition and because of a small sample size, our additional analyses regarding effectiveness have to be interpreted cautiously. Our patients had a statistically significant score reduction in self-reported symptoms (MDI). There was no significant reduction in the HDRS-21 or in the subscale *depression* of the DASS. A reason for a lack of significant result regarding the DASS might be the fact that there were 44% missing data for the weekly filled-out questionnaire. Regarding the HDRS and MDI, it has to be noted that observer-rated instruments benefit from clinician expertise and are argued to be more “objective”, while self-rated questionnaires may capture better subjective experience (33). In a study by Leuchter et al. (34), changes induced with another NIBS, repetitive transcranial magnetic stimulation (rTMS), were better captured by self-report scales. In their study, the HDRS also had the lowest response rates. Nevertheless, the authors stated that a better outcome on a self-report scale might be conceived as a “false positive” benefit with the HDRS as the more accurate measurement (34). Thus, we cannot exclude or determine the extent of placebo effects regarding the MDI data. Available randomized controlled trials of home-based tDCS for depression have not found significant differences in active relative to sham tDCS treatment. Only one single-blinded study by Oh et al. (20) found that active tDCS resulted in a significantly higher reduction of Beck Depression Inventory (BDI-I) scores, which also represents a self-report scale, compared to sham treatment. Therefore, further controlled studies are needed to demonstrate that active home-based tDCS exceeds placebo effects. Nevertheless, half of our patients fulfilled response criteria in all three questionnaires. Our results regarding response rates (MDI: 44.4%) go in line with a study by Alonzo et al. (3) who found a response rate of 38% for observer-rated symptoms (Montgomery Asperg Rating Scale) after 6 weeks of self-administered tDCS stimulation. Another study, by Borrione et al. (17), who used app-based psychological interventions in combination with home-based tDCS, found a response rate (HAMD-17) of 80%. Possible influences (additional app-based intervention, psychiatric medication, etc.) on respective response rates must be taken into account as, e.g., Brunoni et al. (35) found that antidepressants can lead to increased tDCS effects.

Additionally, it is noticeable that non-response was sometimes related to a lower rate of video supervised sessions. This phenomenon might be explained by the positive impact that daily contact with researchers has on depressive symptoms due to social interaction (11). Future sham-controlled studies should consider to investigate the connection between the number of video consultations and depressive outcome in the course of a tDCS treatment.

In the whole sample, physical and psychological quality of life was improved with a large effect size, whereas the environmental and social domain as well as sleep quality remained unchanged. The result regarding the psychological domain goes in line with previous literature that anodal tDCS over left DLPFC improves the processing of positive affective stimuli and reduces the selective attention for negative affective stimuli, thus increasing the psychological domain of life quality (36). An improvement in the physical domain might be correlated with an amelioration of the somatic symptoms of depression, e.g., lack of motivation, over the course of the trial (ICD-10). Home-based tDCS did not improve the social and environmental domains, which might be explained by conducting the treatment at home alone without, e.g., augmented group therapy (37). Moreover, many of our patients forgot the video consultations or implemented further treatment on their own, whereby they had no positive effect from a social interaction with our clinicians. In contrast to a study by Zhou et al. (15) that treated insomnia patients with tDCS at a hospital, improvement of sleep quality was not found in our study (15). The lack of improvement of the sleep quality in our study may be due to the fact that the authors treated patients who suffered from insomnia and thus had worse pre-treatment PSQI scores than ours. Another explanation could be that regular video consultations cannot be compared with controlled sleep times in a sleep laboratory that might have had a positive effect on the sleep quality of the author’s patients. With respect to the follow-up data, we have to notice that more than 50%, mostly non-responders, of our patients were not reachable after termination of the study. Thus, we refrained from an evaluation of the follow-up data because the focus of this study was not on long-lasting antidepressant effects. Study limitations refer to the lack of attrition and/or adherence rates. Future studies should consider including these parameters in order to make potential difficulties regarding the implementation at home statistically comparable.

## Conclusions

Our results show that regular video consultations are needed to ensure good adherence to a predefined protocol (e.g., once a day at 24-h intervals) and to minimize the occurrence of side effects. Nevertheless, in the event that the clinical impression arises that a patient can continue the treatment without further video consultations, other safety concepts should be used in such cases. Furthermore, the present one-armed study on the topic of tDCS at home for depressive disorders provides further evidence regarding usability, tolerability, and effectiveness.

## Data availability statement

The raw data supporting the conclusions of this article will be made available by the authors, without undue reservation.

## Ethics statement

The studies involving humans were approved by Ethikkommission der Universität Regensburg. The studies were conducted in accordance with the local legislation and institutional requirements. The participants provided their written informed consent to participate in this study.

## Author contributions

KD: Formal analysis, Validation, Visualization, Writing – original draft, Writing – review & editing. MA: Investigation, Writing – review & editing. FW: Investigation, Writing – review & editing. MH: Formal analysis, Writing – review & editing. LE: Formal analysis, Writing – review & editing. BL: Conceptualization, Investigation, Project administration, Supervision, Writing – review & editing. TH: Investigation, Writing – review & editing. MS: Conceptualization, Investigation, Project administration, Supervision, Writing – review & editing.
